# *SlERF109-like* and *SlNAC1* Coordinately Regulated Tomato Ripening by Inhibiting *ACO1* Transcription

**DOI:** 10.3390/ijms25031873

**Published:** 2024-02-03

**Authors:** Chen Sun, Gaifang Yao, Jinghan Zhao, Ruying Chen, Kangdi Hu, Guanghua He, Hua Zhang

**Affiliations:** 1 School of Biological and Chemical Engineering, Zhejiang University of Science and Technology, Hangzhou 310012, China; sunchenyeah@163.com (C.S.); sunclab@zust.edu.cn (R.C.); 2 School of Food and Biological Engineering, Hefei University of Technology, Hefei 230009, China; yaogaifang@hfut.edu.cn (G.Y.); jinghanz712@163.com (J.Z.); kangdihu@hfut.edu.cn (K.H.)

**Keywords:** fruit ripening, regulatory complex, *SlACO1*, SlERF109-like-SlNAC1, tomato (*Solanum lycopersicum*)

## Abstract

As a typical climacteric fruit, tomato (*Solanum lycopersicum*) is widely used for studying the ripening process. The negative regulation of tomato fruits by transcription factor *SlNAC1* has been reported, but its regulatory network was unclear. In the present study, we screened a transcription factor, *SlERF109-like*, and found it had a stronger relationship with *SlNAC1* at the early stage of tomato fruit development through the use of transcriptome data, RT-qPCR, and correlation analysis. We inferred that SlERF109-like could interact with SlNAC1 to become a regulatory complex that co-regulates the tomato fruit ripening process. Results of transient silencing (VIGS) and transient overexpression showed that *SlERF109-like* and *SlNAC1* could regulate chlorophyll degradation-related genes (*NYC1*, *PAO*, *PPH*, *SGR1*), carotenoids accumulation-related genes (*PSY1*, *PDS*, *ZDS*), ETH-related genes (*ACO1*, *E4*, *E8*), and cell wall metabolism-related genes expression levels (*CEL2*, *EXP*, *PG*, *TBG4*, *XTH5*) to inhibit tomato fruit ripening. A dual-luciferase reporter and yeast one-hybrid (Y1H) showed that SlNAC1 could bind to the *SlACO1* promoter, but SlERF109-like could not. Furthermore, SlERF109-like could interact with SlNAC1 to increase the transcription for *ACO1* by a yeast two-hybrid (Y2H) assay, a luciferase complementation assay, and a dual-luciferase reporter. A correlation analysis showed that *SlERF109-like* and *SlNAC1* were positively correlated with chlorophyll contents, and negatively correlated with carotenoid content and ripening-related genes. Thus, we provide a model in which SlERF109-like could interact with SlNAC1 to become a regulatory complex that negatively regulates the tomato ripening process by inhibiting *SlACO1* expression. Our study provided a new regulatory network of tomato fruit ripening and effectively reduced the waste of resources.

## 1. Introduction

As a widely cultivated cash crop, tomato (*Solanum lycopersicum*) is rich in many nutrients and it is an essential vegetable for human health. During ripening and senescence, tomato fruits undergo a variety of physiological and biochemical changes, including carotenoid synthesis, chlorophyll degradation, decrease of firmness, and the formation of flavor substances [1]. It is worth noting that the change in color of the tomato fruit from green to red is a visible sign of maturity. This process consists of both chlorophyll degradation and carotenoid biosynthesis. Firstly, chlorophyll was catalyzed by pheophorbide a monooxygenase (PAO), pheophytin pheophorbide hydrolase (PPH), and SGR (STAY-GREEN) protein for degradation [2,3]. Subsequently, the carotenoid was catalyzed by PSY (phytoene synthase), PDS (phytoene desaturase), ZISO (ζ-carotene isomerase), and ZDS (ζ-carotene desaturase) for synthesizing [4]. On the other hand, the decrease of firmness was co-catalyzed by the synergism of many genes in tomato fruit ripening. A large number of enzymes related to cell wall modification could cooperate to hydrolyze the cell wall to soften the textures of tomato fruit. For example, PG (polygalacturonase) and the CEL (hemicellulose/cellulose-modifying enzymes), XTH (xyloglucan transglycosylase/hydrolase) and EXP (expansin) [4,5]. As a typical respiration climacteric fruit, large amounts of ethylene (ETH) are released during the ripening and senescence process of tomatoes [6]. As ETH acts as a plant hormone, it could positively regulate tomato ripening and senescence and directly accelerate the decay of tomato fruit. Thus, the exploration of the tomato ripening mechanism could effectively reduce the waste of resources. 

After decades of research by scientists, the pathway of ETH biosynthesis has gradually become clear. In tomato fruit, ACC (1-aminocyclopropane-1-carboxylic acid) is first produced by ACS (1-aminocyclopropane-1-carboxylic acid synthase), catalyzed by SAM (S-adenosylmethionine), and then ACC is catalyzed by ACO (1-aminocyclopropane-1-carboxylic acid oxidase) oxidase to produce ETH [7,8]. Then, ETH regulated the downstream genes expression by a series of transduction mechanisms to accelerate tomato fruit ripening [9]. Although functions of key genes of the ETH biosynthesis pathway had been identified in tomato, the complex regulatory network of tomato ripening induced by ETH was still unclear. Among the known ETH biosynthetic pathways, there is still a large number of unknown transcription factors (TFs) that also play an important role in fruit ripening and senescence by regulating the expression of key genes of the ETH pathway. Early studies showed that *RIN* (ripening inhibitor) and *NOR* (non-ripening) had been identified as typical TFs that could activate the tomato ripening process [10,11]. *RIN* is a MADS-box transcription factor gene in which there is a deletion of the last exon, and *RIN* plays a positive role in tomato ripening by binding to the promoter of *SlACS2*, *SlACS4*, *SlPG*, *SlTBG4*, *SlEXP1*, and *SlNAC-NOR* [10]. NOR, a member of the NAC domain family, could positively regulate tomato ripening by binding to *SlACO1*, *SlACS1*, and *SlACS2* promoter [11]. On the contrary, NAC domain family transcription factor *SlNAC1* could decrease ETH production in tomato by inhibiting *SlACO1*, *SlACS2*, and *SlPSY1* expression; the tomato fruit with overexpressing *SlNAC1* could be softened while still becoming orange when fully ripened [12]. 

Ethylene response factor (ERF) is a class of transcription factors that is unique to plants. All of the ERF/AP2 superfamily members have an ERF/AP2 domain, which consists of 60 to 70 amino acids and could bind to DNA [13]. Generally, ERFs could bind to the GCC-box (AGCCGCC) and the DRE *cis*-acting element (A/GCCGAC) to play a role in plant physiology [14,15]. In different fruits, several ERFs have been reported to be involved in fruit ripening by directly or indirectly regulating ETH biosynthesis. For instance, an ERF domain family transcription factors APETALA2/ethylene response factor (AP2/ERF) gene, *SlAP2a*, could negatively regulate tomato ripening. RNAi repression of *SlAP2a* fruits showed more carotenoid content and higher expression levels of *SlACO1*, *SlACS2*, and *SlACS4* [16]. *SlERF6* and *SlERF.B3* could directly bind to *SlACO1* and *SlACS2* to regulate ETH production to control tomato fruit [17]. Moreover, *SlERF6* could also be combined with *HSP21* and *DXS* to reduce carotenoid accumulation. In banana, *MaERF11* could inhibit *MaACS1* and *MaACO1* expression by binding promoters, while *MaERF9* could activate *MaACO1* to positively regulate ripening [18,19]. In apples, *MdERF2* and *MdERF3* co-regulate *MdACS1* expression in an antagonistic manner to balance the ripening process in apple [20]. These studies suggested that ERFs played a direct role in controlling the fruit ripening process by regulating the expression of key genes in ETH metabolism. But there are few reports showing that ERFs could interact with other transcription factors to co-regulate fruit development.

To further explore the mechanism of tomato ripening, we screened many transcription factors that had different expression levels at the tomato fruit development stage. We identified the *SlNAC1* and *SlERE109*-*like* as the candidate transcription factors by analyzing the results of RT-qPCR, a correlation analysis, and a dual-luciferase reporter assay. Based on the data obtained, we propose that SlNAC1 could interact with ERF109-like to be a transcription complex to control the tomato fruit ripening process. To validate this hypothesis, we identified the function of SlNAC1 and SlERE109-like in the tomato ripening process using transient silencing (virus-induced gene silencing (VIGS)) and the overexpression of genes. We performed a yeast two-hybrid assay (Y2H) and a luciferase complementation analysis to explore the regulatory relationship between SlNAC1 and SlERF109-like. We also explored the target gene of SlNAC1-SlERF109-like complex using dual-luciferase reporter and yeast one hybrid (Y1H) assays and finally identified the regulatory mechanism. 

## 2. Results

### 2.1. Negative Ripening-Related Regulators Were Screened by Transcriptome Data 

**Figure 1:**
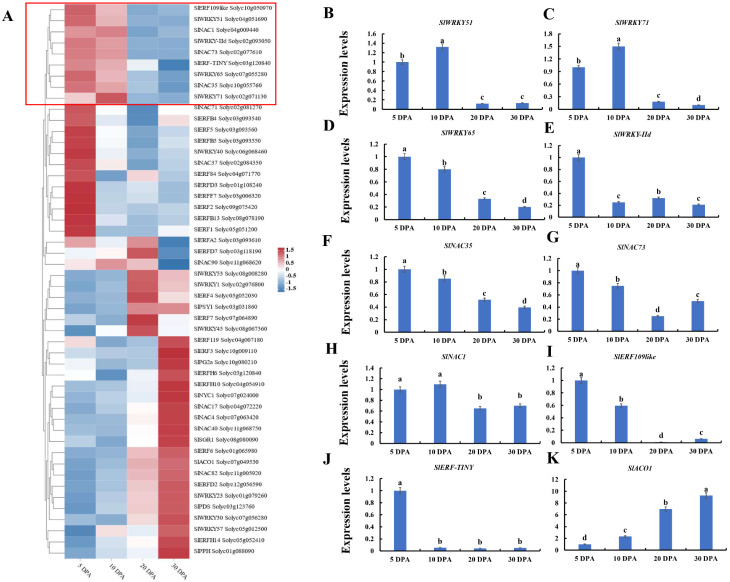
Screening differentially expressed genes (DEGs) from a high-resolution spatiotemporal transcriptome (Shinozaki et al., 2018) [21], and analysis of the expression levels by RT-qPCR. (**A**) Heatmap of the candidate DEGs related to ripening in tomato at 5, 10, 20, and 30 DPA (days post anthesis). The expression levels of (**B**) *SlWRKY51*, (**C**) *SlWRKY71*, (**D**) *SlWRKY65*, (**E**) *SlWRKY-IId*, (**F**) *SlNAC35*, (**G**) *SlNAC73*, (**H**) *SlNAC1*, (**I**) *SlERF109like*, (**J**) *SlERF-TINY*, and (**K**) *ACO1* were analyzed using Rt-qPCR in tomato fruits at 5, 10, 20, and 30 DPA. Data are presented as means ± SD (*n* = 3). Different letters above the columns stand for significant differences between two values (*p* < 0.05) at the same time point.

The process of fruit ripening and senescence is an extremely complex regulatory network and a large number of unknown genes and transcription factors are involved in this process. A recent study on the transcriptome analysis of different tissues of tomato fruit provides new insights into the process of ripening and senescence in tomato fruit [21]. Based on transcriptome analysis, we screened and obtained some ripening-related genes, including *SlACO1*, *SlPPH*, *SlPSY1*, *SlPDS*, *SlSGR1*, and *SlPG2a*, differentially expressed at 5 DPA (days post anthesis), 10 DPA, 20 DPA, and 30 DPA. We also screened some transcription factors that had high expression at 5 DPA and 10 DPA, which then decreased at 20 DPA and 30 DPA, including *SlERF109-like*, *SlERF-TINY*, *SlNAC1*, *SlNAC73*, *SlNAC35*, *SlWRKY71*, *SlWRKY51*, *SlWRKY65*, and *SlWRKYII-d*. This suggested that these transcription factors may play a negative role in the fruit ripening process (Figure 1A). By analyzing the RT-qPCR results, *SlERF109-like*, *SlERF-TINY*, *SlNAC1*, *SlNAC73*, *SlNAC35*, *SlWRKY71*, *SlWRKY51*, *SlWRKY65*, and *SlWRKYII-d* had higher expression levels in the early stage (5 to 10 DPA) of tomato fruit development, while they had lower expression levels at the late stage of tomato ripening (Figure 1B–J). On the contrary, the expression level of *SlACO1* was rising as the tomato fruit ripens (Figure 1K). These results suggested that *SlERF109-like*, *SlERF-TINY*, *SlNAC1*, *SlNAC73*, *SlNAC35*, *SlWRKY71*, *SlWRKY51*, *SlWRKY65*, and *SlWRKYII-d* could play a negative role in the tomato fruit ripening process. 

### 2.2. Identification of the Regulatory Relationship between the Candidate Transcription Factors and ACO1

**Figure 2:**
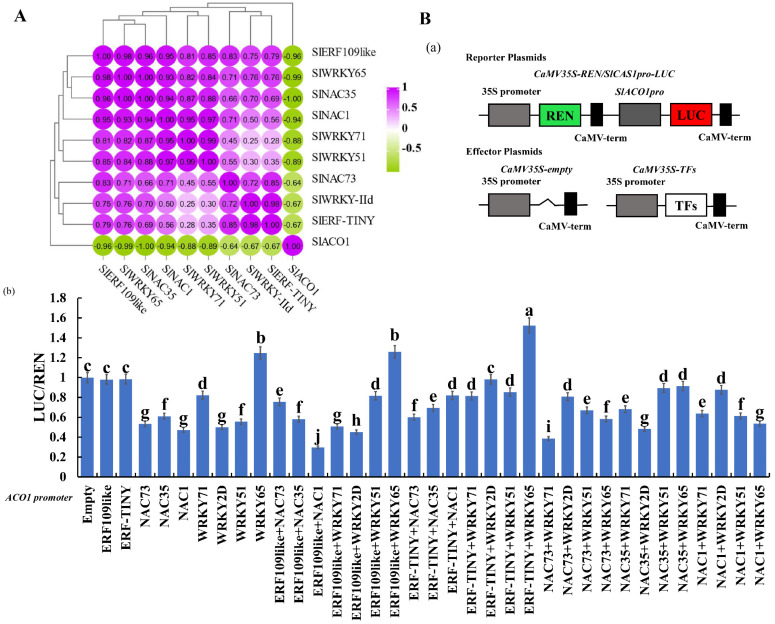
Identification of transcription factors regulating *SlACO1* transcription in tomato fruit. (**A**) Correlation analysis of *SlERF109-like*, *SlWRKY65*, *SlNAC35*, *SlNAC1*, *SlWRKY71*, *SlWRKY51*, *SlNAC73*, *SlWRKY-IId*, *SlERF-TINY*, and *SlACO1* expression. (**B**) Transactivation activity of TFs for the *SlACO1* promoter. (**a**) Model of reporter and effector plasmids. (**b**) TFs activating the *SlACO1* promoter were validated with the dual-luciferase reporter assay. Data are presented as means ± SD (*n* = 3). Different letters above the columns stand for significant differences between two values (*p* < 0.05) at the same time point.

To determine the relationship between candidate transcription factors and *SlACO1*, we performed a correlation analysis of their expressions. As shown in Figure 2A, *SlERF109-like* (*pearson*: −0.96), *SlERF-TINY* (*pearson*: −0.67), *SlNAC1* (*pearson*: −0.94), *SlNAC73* (*pearson*: −0.64), *SlNAC35* (*pearson*: −1), *SlWRKY71* (*pearson*: −0.88), *SlWRKY51* (*pearson*: −0.89), *SlWRKY65* (*pearson*: −0.99), and *SlWRKYII-d* (*pearson*: −0.67) were negatively correlated with *SlACO1*. This suggested that these candidate transcription factors were potential repressors for *SlACO1*. To further identify the transactivation of regulators for *SlACO1*, we conducted a dual-luciferase reporter assay of the *SlACO1* promoter and TFs by tobacco leaves. As shown in Figure 2B, *SlNAC1*, *SlNAC73*, *SlNAC35*, *SlWRKY71*, *SlWRKYII-d*, and *SlWRKY51* could inhibit *SlACO1* compared with empty vectors, while *SlWRKY65* could activate the *SlACO1* promoter (*p* < 0.05). Interestingly, after grouping these transcription factors and then co-transforming them with the promoter of *SlACO1* into tobacco leaves, we found that *SlERF109-like* could significantly inhibit the transactivation of *SlNAC1* to *SlACO1* (*p* < 0.05).

### 2.3. Identification and Bioinformatics Analysis of SlERF109-like in Plant

**Figure 3:**
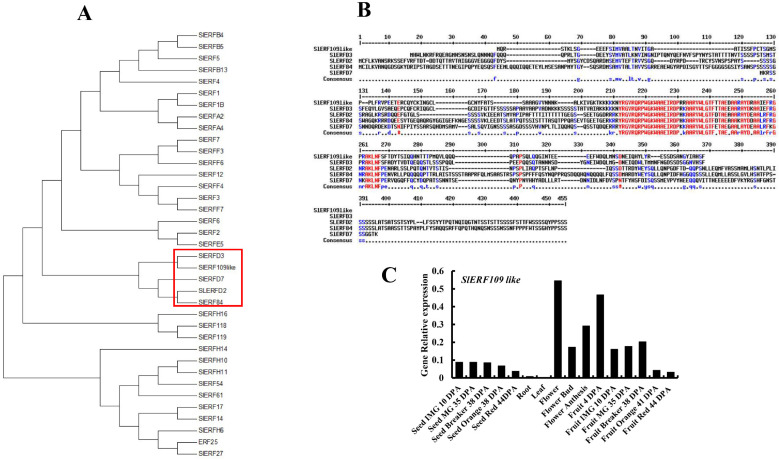
Identification and bioinformatics analyses of ERF109-like in different plants. (**A**) The ERF genes of tomatoes are analyzed in the phylogenetic tree. The analysis was performed using the neighbor-joining method by the MEGA7 Program. (**B**) Amino acid sequence analysis of *SlERFD2*, *SlERFD3*, SlERFD7, *SlERF84*, and *SlERF109-like* (http://multalin.toulouse.inra.fr/multalin/ (accessed on 8 May 2023)). (**C**) The transcription of *SlERF109-like* genes in different tissues of the tomato is analyzed by public data. The original expression data were obtained from the online tool, http://tomexpress.toulouse.inra.fr/query (accessed on 15 May 2023), and the tomato cultivar was Micro Tom. Data are presented as means ± SD (*n* = 3).

Then, we analyzed the evolutionary relationships of *SlERF109-like* by constructing an evolutionary tree, and the results showed that *SlERF109-like* was highly homologous to *SlERFD3*, *SlERF84*, and *SlERFD2* (Figure 3A). Sequence analysis results showed that all of *SlERF109-like*, *SlERFD3*, *SlERF84*, and *SlERFD2* had a conservative AP2 domain (Figure 3B). These results suggested that *SlERF109-like* had ERF family functions and could be involved in the plant developmental process. Moreover, we searched the transcription data of *SlERF109-like* in tomato roots, stems, leaves, flower buds, flowers, and fruits at different ripening stages in a public database (http://tomexpress.toulouse.inra.fr/ (accessed on 28 May 2023)) to explore the expression patterns in tomato plants. As shown in Figure 3C, there were higher expressions of *SlERF109-like* in the flowers and fruits of the tomato plant. The expression of *SlERF109-like* was higher when the fruit was at the early stage of development (4 DPA), but gradually decreased with fruit ripening (10 to 38 DPA) and reached the lowest value at full maturity (44 DPA). This is consistent with our previous RT-qPCR analysis of the expression pattern of *SlERF109-like* in tomatoes at 5 DPA, 10 DPA, 20 DPA, and 30 DPA.

### 2.4. Identifying the Function of SlNAC1 and SlERF109-like in Tomato Fruit by VIGS

**Figure 4:**
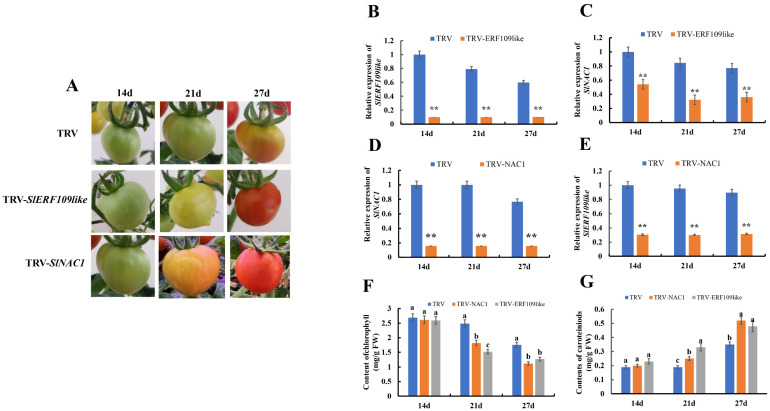
Transient silencing expression of *SlNAC1* and *SlERF109-like* on the ripening process of tomatoes using VIGS. (**A**) Phenotype of *SlNAC1*-silenced (TRV-*SlNAC1*) and *SlERF109-like*-silenced (TRV-*SlERF109like*) fruit in tomatoes at 14, 21, and 27 days post-infection. Expression levels of (**B**) *SlERF109-like* and (**C**) *SlNAC1* in *SlERF109-like*-silenced tomato fruits. Expression levels of (**D**) *SlNAC1* and (**E**) *SlERF109-like* in *SlNAC1*-silenced tomato fruits. (**F**) Contents of chlorophyll in TRV-*SlNAC1*, TRV-*SlERF109-like*, and the control group (TRV). (**G**) Contents of carotenoids in TRV-*SlNAC1*, TRV-*SlERF109-like*, and the control group (TRV) fruits. Data are presented as means ± SD (*n* = 3). Different letters (*p* < 0.05) or ** (*p* < 0.01) above the columns stand for significant differences between two values at the same time point.

To investigate the role of *SlNAC1* and *SlERF109-like* in regulating tomato ripening, we silenced *SlERF109-like* and *SlNAC1* in tomato fruit using VIGS, respectively. As shown in Figure 4A, we recorded phenotypic changes in tomatoes at 14, 21, and 27 days post infection (DPI). We found that TRV-*SlERF109-like* fruits changed to yellow at 21 DPI, while the control group was still maintaining a green color at 21 DPI. At 27 DPI, TRV-*SlERF109-like* fruits became red, whereas the control group fruits had just completed the change from green to yellow. Through an analysis of *SlERF109-like* expression of TRV-*SlERF109-like* and a control group, we found that the expression of *SlERF109-like* was significantly suppressed, which suggested that we had successfully silenced *SlERF109-like* using VIGS (Figure 4B). Moreover, we detected the expression level of *SlERF109-like*-interaction factor *SlNAC1* and found that it decreased in TRV-*ERF109-like* tomato fruits (Figure 4C).

Similarly, the phenotype of TRV-*SlNAC1* was observed at 14, 21, and 27 DPI. At 21 DPI, the TRV-*SlNAC1* fruits became orange while the control group fruits were still green. When the TRV-*SlNAC1* fruits had finished changing to red, the control group fruits were still yellow (Figure 4A). Through the RT-qPCR analysis, we found that the *SlNAC1* expression level was decreased in TRV-*SlNAC1* tomato fruit compared with that of the control group, suggesting that *SlNAC1* had been successfully silenced by VIGS (Figure 4D). The expression level of the *SlNAC1*-interaction factor, *SlERF109-like*, also decreased, as *SlNAC1* was silenced, compared with the control group (Figure 4E). 

Color change is an important sign of the tomato ripening process, and it is led by the degradation of chlorophyll and the accumulation of carotenoids. To explore the mechanism of *SlERF109-like* and *SlNAC1* silencing and transient expression in the tomato ripening process, we determined the contents of chlorophyll and carotenoids in the control group, the TRV-*SlNAC1* group, and the TRV-*SlERF109-like* group. As shown in Figure 4F, the chlorophyll content decreased in the control, TRV-*SlNAC1*, and TRV-*SlERF109-like* groups at 21 and 27 DPI, and the chlorophyll content in the control group was 1.5 times that of the TRV-*SlNAC1* and TRV-*SlERF109-like* groups (*p* < 0.05). The carotenoids in the control, TRV-*SlNAC1*, and TRV-*SlERF109-like* groups increased at 21 and 27 DPI, and the carotenoids content in both TRV-*SlNAC1* and TRV-*SlERF109-like* groups was 1.5 times that of the control group (*p* < 0.05) (Figure 4G). 

### 2.5. Effect of Gene Silencing of SlNAC1 and SlERF109-like on the Expression of Ripening-Related Genes

**Figure 5:**
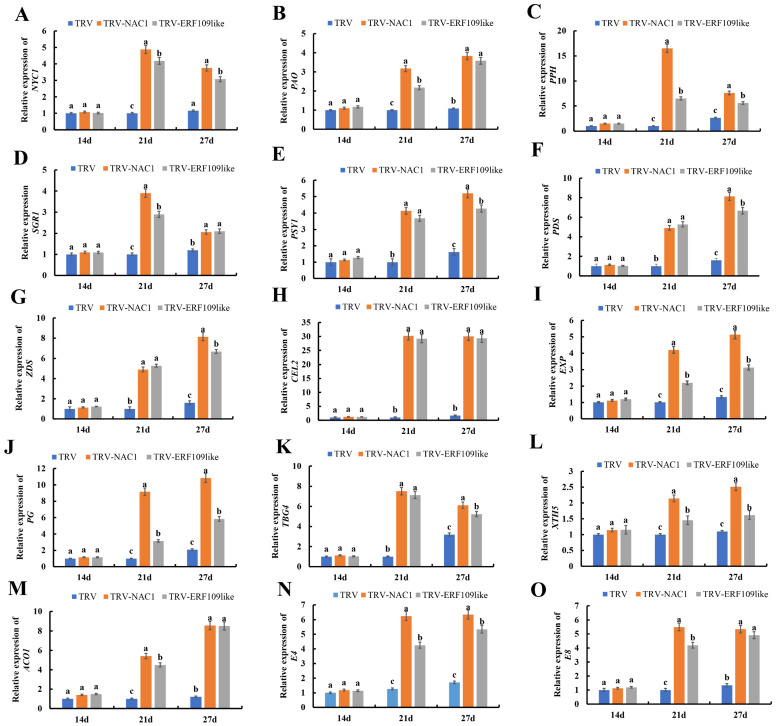
Expression levels of (**A**) *NYC1*, (**B**) *PAO*, (**C**) *PPH*, (**D**) *SGR1* (**E**) *PSY1*, (**F**) *PDS*, (**G**) *ZDS*, (**H**) *CEL2*, (**I**) *EXP*, (**J**) *PG*, (**K**) *TBG4*, (**L**) *XTH5*, (**M**) *ACO1*, (**N**) *E4*, and (**O**) *E8* in TRV-*SlNAC1*, TRV-*SlERF109-like*, and control group (TRV) fruits. Data are presented as means ± SD (*n* = 3). Different letters above the columns stand for significant differences between two values (*p* < 0.05) at the same time point.

To explore the mechanism of the differences in the chlorophyll content in the control group, the TRV-*SlNAC1* group, and the TRV-*SlERF109-like* group, we analyzed the expression levels of *NYC1*, *PAO*, *PPH*, and *SGR1* using RT-qPCR to analyze the chlorophyll degradation pathway. As shown in Figure 5A–D, the expression levels of *NYC1*, *PAO*, *PPH*, and *SGR1* increased in all groups—TRV-*SlNAC1*, and TRV-*SlERF109-like*, and the control group—at 21 DPI and 27 DPI, and these expression levels in both the TRV-*SlNAC1* and TRV-*SlERF109-like* groups were nearly 6 times higher than those in the control group (*p* < 0.05). 

Carotenoids accumulation is an important character of tomato ripening. We determined expression levels of key genes, including *PSY1*, *PDS*, and *ZDS*, in the carotenoids biosynthesis pathway in order to analyze the mechanism of differences in the carotenoids content in the control group, and in the TRV-*SlNAC1* and *SlERF109-like* fruits. As shown in Figure 5E–G, expression levels of *PSY1*, *PDS*, and *ZDS* increased in TRV-*SlNAC1*, TRV-*SlERF109-like*, and control groups at 21 and 27 DPI. The expression levels of *PSY1*, *PDS*, and *ZDS* in TRV-*SlNAC1* and TRV-*SlERF109-like* were over five times higher than those in the control group (*p* < 0.05), suggesting that silencing *SlNAC1* or *SlERF109-like* could lead to carotenoids accumulation by increasing *PSY1*, *PDS*, and *ZDS* expression. 

Usually, a decrease in fruit firmness happens with the fruit ripening process and it is always regulated by genes or enzymes related to cell wall metabolism. Thus, we analyzed the key genes that encode cell wall metabolism-related enzymes, including *CEL2*, *EXP*, *PG*, *TBG4*, and *XTH5*. The RT-qPCR results showed that *CEL2*, *EXP*, *PG*, *TBG4*, and *XTH5* expression levels were increased in the control group, and in TRV-*SlNAC1* and TRV-*SlERF109-like* tomato fruits at 21 and 27 DPI (Figure 5H–L), while the expression levels of *CEL2*, *EXP*, *PG*, *TBG4*, and *XTH5* in TRV-*SlNAC1* and TRV-*SlERF109-like* tomato fruits were always much higher than those of the control group. For instance, the expression of *CEL2* in TRV-*SlNAC1* and TRV-*SlERF109-like* was 30 times and 28 times higher than that of the control group fruits, respectively (Figure 5H). 

We also determined ethylene-related expression in the control, TRV-*SlNAC1*, and *SlERF109-like* groups, to explore the mechanism of the differences in tomato ripening. As shown in Figure 5M, the expression levels of *SlACO1* increased in the control, TRV-*SlNAC1*, and TRV-*SlERF109-like* groups at 21 and 27 DPI. The expression levels of *SlACO1* in TRV-*SlNAC1* and TRV-*SlERF109-like* were 6 to 10 times higher than those in the control group. This suggested that *SlNAC1* and *SlERF109-like* could decrease *SlACO1* transcription. Moreover, E4 (encoding the peptide methionine sulfoxide reductase msrA) and E8 (1-aminocyclopropane-1-carboxylate oxidase-like protein) are important ethylene-responsive marker genes in tomato; the expression levels of *E4* and *E8* could reflect the degree of tomato ripeness. By analyzing the expression levels of *E4* and *E8*, we found that *E4* and *E8* expression levels are five to seven times higher in TRV-*SlNAC1* and TRV-*SlERF109-like* tomato fruits than those in the control group (*p* < 0.05) (Figure 5N,O). This indicates that the tomato fruits of TRV-*SlNAC1* and TRV-*SlERF109-like* were more mature than those of the control group. 

### 2.6. Identifying the Function of SlNAC1 and SlERF109-like in Tomato Fruit by Transient Overexpression

**Figure 6:**
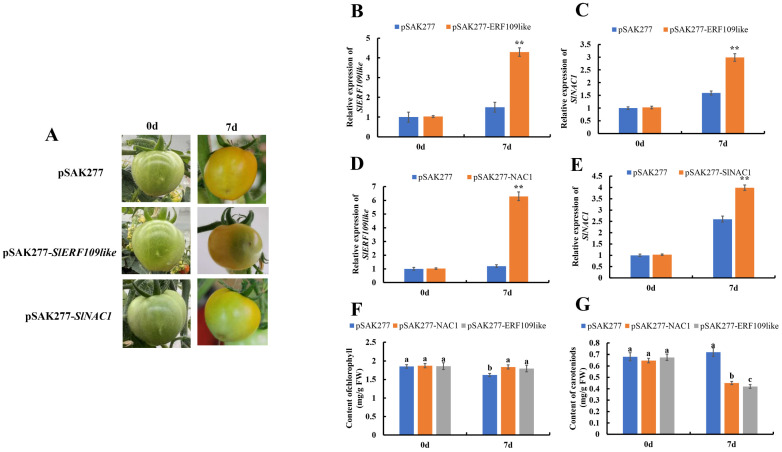
Transient overexpression of *SlNAC1* and *SlERF109-like* in the ripening process of tomato by pSAK277-*SlNAC1* and pSAK277-*SlERF109-like*. (**A**) Phenotype of *SlNAC1*-overexpressed (pSAK277-*SlNAC1*) and *SlERF109-like*-overexpressed (pSAK277-*SlERF109llike*) tomato fruit. Expression levels of (**B**) *SlERF109-like* and (**C**) *SlNAC1* in pSAK277-*SlERF109-like* tomato fruits. Expression levels of (**D**) *SlERF109-like* and (**E**) *SlNAC1* in pSAK277-*SlNAC1* tomato fruits. (**F**) Contents of chlorophyll in pSAK277-*SlNAC1*, pSAK277-*SlERF109-like*, and the control group (pSAK277). (**G**) Contents of carotenoids in pSAK277-*SlNAC1*, pSAK277-*SlERF109-like*, and the control group (pSAK277). Different letters (*p* < 0.05) or ** (*p* < 0.01) above the columns stand for significant differences between two values at the same time point.

To comprehensively investigate the role of *SlNAC1* and *SlERF109-like* in regulating tomato ripening, we performed transient over-expression in tomato fruits using pSAK277-*NAC1*/*ERF109like*. As shown in Figure 6A, the tomato fruits ripened later than in the control group by infecting with pSAK277-*SlERF109-like* at 0 to 7 DPI. The expression level of *SlERF109-like* in pSAK277-*SlERF109-like* was shown to increase significantly through an RT-qPCR analysis (Figure 6B). The expression level of SlNAC1 also increased in pSAK277-*SlERF109-like* tomato fruits (Figure 6C). 

Contrary to TRV-*SlNAC1* fruits, the tomato fruits infected by pSAK277-*SlNAC1* showed a ripening-late phenotype compared with the control group. At 7 DPI, the color of control group fruits began to change to yellow while pSAK277-*SlNAC1* were still green (Figure 6A). RT-qPCR analysis showed that *SlNAC1* expression increased by 6-fold, which was confirmed in pSAK277-*SlNAC1* fruits, indicating that *SlNAC1* expressed successfully (Figure 6D). *SlERF109-like* expression level was also determined to have increased as *SlNAC1* expression increased in pSAK277-*SlNAC1* (Figure 6E). The above results suggested that *SlERF109-like* and *SlNAC1* could play a negative role in tomato fruit ripening. 

We also determined the contents of chlorophyll and carotenoids in the control group, the pSAK277-*SlNAC1* group, and the pSAK277-*SlERF109-like* group. As shown in Figure 6F, the chlorophyll content in both pSAK277-*SlNAC1* and pSAK277-*SlERF109-like* was higher than that of the control group at 7 DPI, while the carotenoids content in both pSAK277-*SlNAC1* and pSAK277-*SlERF109-like* was significantly lower than that of the control group (Figure 6G). These data were consistent with the phenotypic results. 

### 2.7. Effect of Gene Overexpression of SlNAC1 and SlERF109-like on the Expression of Ripening-Related Genes

**Figure 7:**
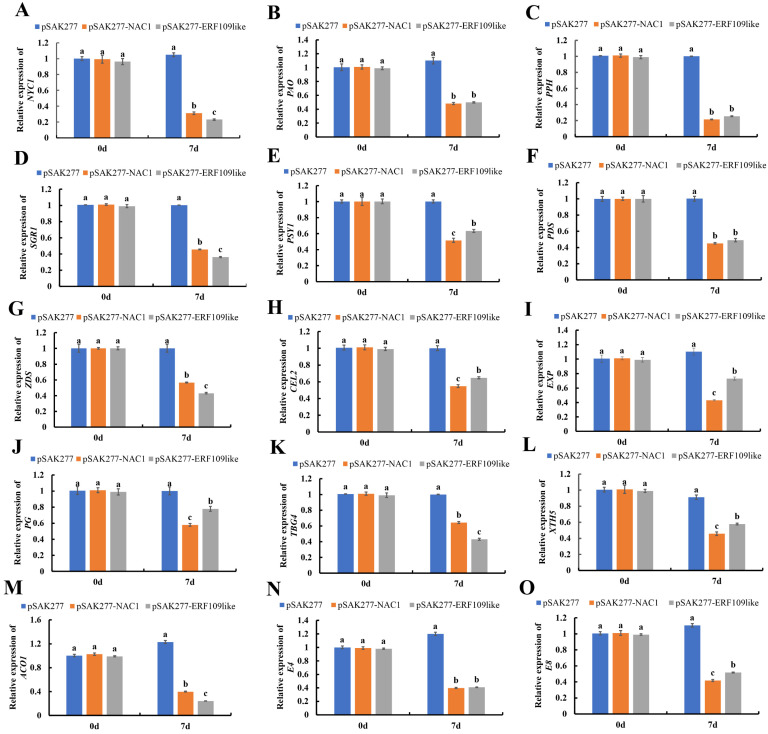
Expression levels of (**A**) *NYC1*, (**B**) *PAO*, (**C**) *PPH*, (**D**) *SGR1* (**E**) *PSY1*, (**F**) *PDS*, (**G**) *ZDS*, (**H**) *CEL2*, (**I**) *EXP*, (**J**) *PG*, (**K**) *TBG4*, (**L**) *XTH5*, (**M**) *ACO1*, (**N**) *E4*, and (**O**) *E8* in pSAK277-*SlNAC1*, pSAK277-*SlERF109-like*, and control group (pSAK277). Data are presented as means ± SD (*n* = 3). Different letters above the columns stand for significant differences between two values (*p* < 0.05) at the same time point.

As shown in Figure 7A–D, the expression levels of *NYC1*, *PAO*, *PPH*, and *SGR1* in both pSAK277-*SlNAC1* and pSAK-*SlERF109-like* were much lower than those of the control group at 7 DPI. For instance, the expression levels of *PPH* in the control group were three times those of both pSAK277-*SlNAC1* and pSAK-*SlERF109-like* fruits (*p* < 0.05) (Figure 7C). This indicated that *SlNAC1* and *SlERF109-like* could slow down chlorophyll degradation, possibly by decreasing the key gene expression levels in the chlorophyll degradation pathway. 

Similarly, as shown in Figure 7E–G, expression levels of *PSY1*, *PDS*, and *ZDS* in the control group were nearly two times those of both pSAK277-*SlNAC1* and pSAK277-*SlERF109-like* fruits at 7 DPI (*p* < 0.05), suggesting that overexpressing *SlNAC1* or *SlERF109-like* could slow down the carotenoids accumulation by decreasing *PSY1*, *PDS*, and *ZDS* expression. 

We also analyzed the key genes that encode cell wall metabolism-related enzymes, including *CEL2*, *EXP*, *PG*, *TBG4*, and *XTH5*. As shown in Figure 7H–L, the expression levels of *CEL2*, *EXP*, *PG*, *TBG4*, and *XTH5* were lower in TRV-*SlNAC1* and TRV-*SlERF109-like* than those of the control group fruits at 7 DPI (*p* < 0.05). These data suggested that transient expressed *SlNAC1* and *SlERF109-like* could delay the tomato fruit softening process.

By determining the ETH-related gene expression levels, we found that *ACO1* was nearly three to four times higher in the control group than in the pSAK277-*SlNAC1* and pSAK277-*SlERF109-like* fruits (*p* < 0.05) (Figure 7M). This suggested that over-expressing *SlNAC1* and *SlERF109-like* inhibited *SlACO1* transcription. Moreover, as shown in Figure 7N,O, *E4* and *E8* expression levels were much lower in both pSAK277-*SlERF109-like* and pSAK277-*SlNAC1* fruits than in the control group (*p* < 0.05). This suggested that the ripeness of both pSAK277-*SlNAC1* and pSAK277-*SlERF109-like* tomato fruit was lower than that of the control group; this is consistent with the phenotype of pSAK277-*SlNAC1* and pSAK277-*SlERF109-like* tomato fruit.

### 2.8. SlERF109-like Could Interact with SlNAC1 to Form a Regulatory Complex 

**Figure 8:**
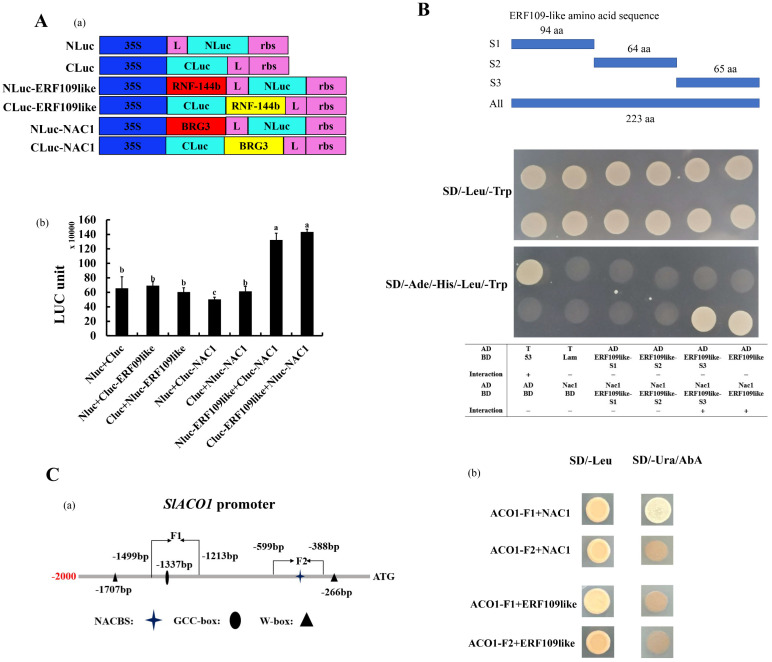
Identification of regulatory relationship between *SlNAC1* and *SlERF109-like*. (**A**) Interaction between *SlNAC1* and *SlERF109-like*. (**a**) Model of nLuc/cLuc, *SlNAC1*-nLuc/cLuc and *SlERF109-like*-nLuc/cLuc constructs. (**b**) Firefly luciferase complementation assays of nLuc/cLuc, *SlNAC1*-nLuc/cLuc, and *SlERF109-like*-nLuc/cLuc in six-week-old tobacco leaves. (**B**) Y2H assay of interactions between *SlNAC1* and *SlERF109-like*. Positive colonies signify strong interactions between *SlNAC1* and *SlERF109-like*. (**C**) *SlNAC1* can bind directly to the *SlACO1* promoter while *SlERF109-like* cannot. (**a**) Scheme of the promoter of *SlACO1*. Bar segments were analyzed using the PlantCARE database and are separated by solid lines (F1, F2). (**b**) Identification of *SlNAC1* bound to the *SlACO1* promoter with the yeast one-hybrid assay.

In order to verify the suspicion that SlERF109-like could interact with SlNAC1, we inserted *SlERF109-like* and *SlNAC1* into Nluc and Cluc vectors, respectively, for luciferase complementation experiments. The results showed that there was a strong interaction between SlNAC1 and SlERF109-like (Figure 8A). Meanwhile, the Y2H assay also showed a similar result, that SlERF109-like-S3 is the region that could interact with SlNAC1 in yeast (Figure 8B). To verify the regulatory mechanism among SlERF109-like, SlNAC1, and *SlACO1*, we performed a Y1H assay and the result showed that SlNAC1 could directly bind to the *SlACO1* promoter by way of the NAC binding site, while SlERF109-like cannot (Figure 8C). 

### 2.9. Correlation Analysis Different Physiological Indexes and Ripening-Related Gene Expression 

**Figure 9:**
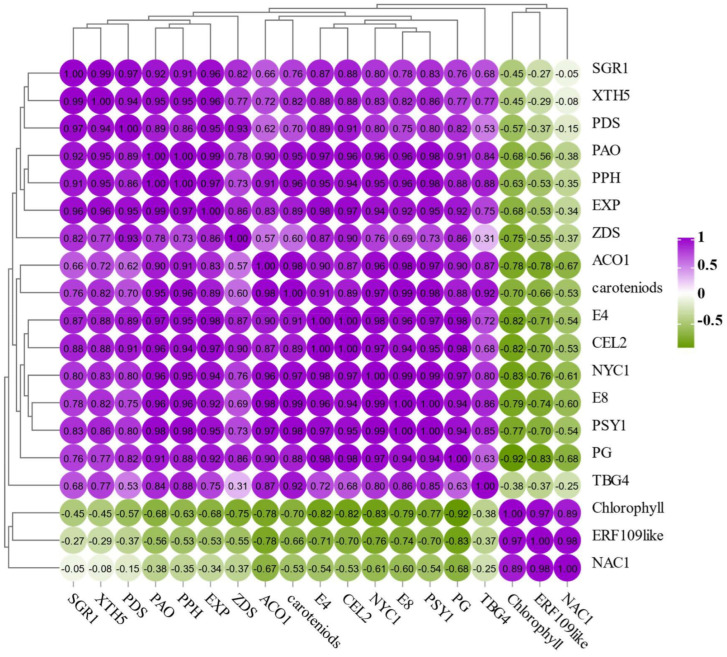
Correlation analysis of chlorophyll, carotenoid contents, and gene expressions of *NYC1*, *PAO*, *PPH*, *SGR1*, *PSY1*, *PDS*, *ZDS*, *E4*, *ACO1*, *E8*, *SlNAC1*, *SlERF109-like*, *PG*, *XTH5*, *TBG4*, *EXP*, and *CEL2*, based on control (TRV) and *SlERF109-like*-silenced fruit (TRV-*SlERF109-like*) at 21 and 27 DPI, and on control (pSAK277) and *SlERF109-like*-overexpressed (pSAK277-*SlERF109-like*) fruit at 7 DPI.

To analyze the potential regulatory relationship between *SlERF109-like*, *SlNAC1*, and tomato fruit ripening, we performed a correlation analysis of the expression levels of *SlERF109-like*, *SlNAC1*, *NYC1*, *PAO*, *PPH*, *SGR1*, *PSY1*, *PDS*, *ZDS*, *ACO1*, *E4*, *E8*, *PG*, *XTH5*, *TBG4*, *EXP*, *CEL2*, and chlorophyll and carotenoids contents. As shown in Figure 9, *SlERF109-like* had a positive correlation with *SlNAC1* (*pearson*: 0.98) and chlorophyll content (*pearson*: 0.97), while it was negatively correlated with the carotenoids contents (*pearson*: −0.66), *NYC1* (*pearson*: −0.76), *PAO* (*pearson*: −0.56), *PPH* (*pearson*: −0.53), *SGR1* (*pearson*: −0.27), *PSY1* (*pearson*: −0.70), *PDS* (*pearson*: −0.37), *ZDS* (*pearson*: −0.55), *ACO1* (*pearson*: −0.78), *E4* (*pearson*: −0.71), *E8* (*pearson*: −0.74), *PG* (*pearson*: −0.83), *XTH5* (*pearson*: −0.29), *TBG4* (*pearson*: −0.37), *EXP* (*pearson*: −0.53), and *CEL2* (*pearson*: −0.70). This suggested that *SlERF109-like* is a negative regulator of tomato fruit ripening. Additionally, SlNAC1 was also positively correlated with chlorophyll contents (*pearson*: 0.89) and negatively correlated with carotenoids contents (*pearson*: −0.53), *NYC1* (*pearson*: −0.61), *PAO* (*pearson*: −0.38), *PPH* (*pearson*: −0.35), *SGR1* (*pearson*: −0.05), *PSY1* (*pearson*: −0.54), *PDS* (*pearson*: −0.15), *ZDS* (*pearson*: −0.37), *ACO1* (*pearson*: −0.67), *E4* (*pearson*: −0.54), *E8* (*pearson*: −0.60), *PG* (*pearson*: −0.68), *XTH5* (*pearson*: −0.08), *TBG4* (*pearson*: −0.25), *EXP* (*pearson*: −0.34), and *CEL2* (*pearson*:−0.53) (Figure 9). Moreover, the ETH-related genes, including *ACO1*, *E4*, and *E8*, were positively correlated with chlorophyll degradation-related genes, cell wall metabolism-related genes, and carotenoids biosynthesis-related genes. This indicated that ETH could play an important role in tomato ripening by regulating chlorophyll degradation and metabolism-related and carotenoids biosynthesis pathways.

## 3. Discussion

As an important plant hormone, ethylene (ETH) could accelerate plant development. Fruit ripening has been widely studied in recent decades [9]. It has been confirmed that SAM is converted to ACC by ACS and then by ACO to ETH in the ETH biosynthesis pathway. The ACSs and ACOs have been indicated as the key rate-limiting enzymes of ETH biosynthesis [7,8]. However, the gene network is huge and complex, and a large number of transcription factors also mediate the tomato ripening process by regulating key genes in the ETH biosynthesis pathway, but the regulatory mechanism is still unclear. *RIN* (ripening inhibitor) and *NOR* (non-ripening) are known to be important transcription factors that can regulate tomato fruit ripening by binding to the ACOs and ACSs promoters [10,11]. Besides *RIN* and *NOR*, many more transcription factors have been studied and are reported to be involved in the regulation of the fruit ripening process, including NACs, WRKYs, and ERFs etc. [12,17,18,19,22,23]. 

A recent study showed several differential expression transcription factors were identified in tomato fruits [21]. In our study, we screened nine factors, including *SlERF109-like*, *SlERF-TINY*, *SlNAC1*, *SlNAC73*, *SlNAC35*, *SlWRKY71*, *SlWRKY51*, *SlWRKY65*, and *SlWRKYII-d*, which were more highly expressed during the early development stage in tomato fruit, and the expression levels decreased as the fruit ripened. Meanwhile, all of these nine transcription factors had a strong negative correlation with SlNAC1 and ACO1, indicating that they might be involved in tomato ripening process (Figure 1 and Figure 2). Results of dual-luciferase reporter showed *SlNAC1* could activate *ACO1* transcription, and *SlERF109*-*like* could enhance the activation effect of *SlNAC1* on *ACO1* (Figure 2B). It suggested that *SlNAC1* with *SlERF109-like* might be involved in the tomato ripening by regulating *ACO1*. 

The APETALA2/ethylene response factor (AP2/ERF) superfamily often played a role in ethylene biosynthesis, fruit color change [17,24], softening [18,25], and flavor [26]. Evolutionary tree analysis showed that *ERF109like* had high homology with *SlERFD2*, *SlERF84*, *SlERFD3*, and *SlERFD7* (Figure 3C), in which *SlERFD7* could be involved in the tomato ripening process by regulating ethylene biosynthesis and signaling, lycopene biosynthesis, and cell wall metabolism [27]. Transient silencing of *SlERF109-like* in tomato fruit showed that the ripening process was accelerated, and the transient overexpression of *SlERF109-like* showed the opposite phenotype (Figure 4A and Figure 6A), as did the chlorophyll and carotenoids contents in the TRV-*SlERF109-like* and pSAK277-*SlERF109-like* tomato fruit (Figure 4C,D and Figure 6C,D). The expression levels of ripening-related genes, including *NYC1*, *PAO*, *PPH*, *SGR1*, *PSY1*, *PDS*, *ZDS*, *CEL2*, *EXP*, *XTH5*, *PG*, *TBG4*, *ACO1*, *E4*, and *E8* were increased in TRV-*SlERF109-like* and decreased in pSAK277-*SlERF109-like*, indicating that *SlERF109-like* is a negative regulator of the tomato ripening process (Figure 5 and Figure 7). ERFs are considered to be important components of the ethylene-responsive mechanism, which could mediate the ripening process. For instance, it was reported that *SlERF*.*B3* could directly regulate ETH biosynthesis-related genes to control tomato fruit ripening, and ERFs also could be the target genes of *SlERF*.*B3* [24]. *SlERFD7* could directly regulate *SlARF2A* and *SlARF2B* amalgamate auxin and ethylene signaling pathways to mediate tomato fruit ripening [27]. *SlERF. F12* could delay tomato ripening by interacting with co-repressor *TOPLESS* and histone deacetylases to target the regulation of *ACS2* and *ACS4* [28]. These studies suggest that ERFs could directly regulate the ripening-related genes expression, and could also mediate other pathways to indirectly affect the tomato ripening process. 

A previously study showed that *SlNAC1* acted as a negative regulator inhibiting the tomato fruit ripening process by down-regulating *SlACO1* expression and decreasing ETH production [12]. The phenotype of tomato fruit infected by TRV-*SlNAC1* also showed that the ripening process was accelerated, and the chlorophyll degradation and carotenoids accumulation increased (Figure 5), but pSAK277-SlNAC1 showed the opposite phenotype (Figure 7). The expression levels of ripening-related genes were accelerated in TRV-*SlNAC1* tomato fruits and decreased in pSAK277-*SlNAC1* tomato fruits (Figure 6 and Figure 8). Notably, the expression levels of ETG-related genes, including *ACO1*, *E4*, and *E8* were increased in TRV-*SlNAC1* tomato fruits and decreased in pSAK277-*SlNAC1* tomato fruits (Figure 8). This is consistent with Ma et al. (2014) [12], who suggested that *SlNAC1* could regulate *ACO1* expression to mediate tomato ripening. Previously, *SlNAC4* was reported to interact with *SlRIN*, *SlNOR*, and *SlERF4* at the protein level and affect *ACS2*, *ACS4*, *ACO1*, and *ACO3* expression [29]. In our study, when overexpressing or silencing *SlERF109*-*like*, the expression of *SlNAC1* also changed. The reverse is also true (Figure 4 and Figure 6). The results of Y2H and luciferase complementation assays showed that SlERF109-like could interact with SlNAC1 at the protein level (Figure 8A,B). Moreover, the Y1H and dual-luciferase reporter assays showed that only *SlNAC1* could bind to the *ACO1* promoter while *SlERF109-like* cannot (Figure 2 and Figure 8C). These data indicated that *SlERF109-like* with *SlNAC1* formed a regulatory complex to co-regulate *ACO1* transcription, thereby inhibiting tomato ripening.

**Figure 10:**
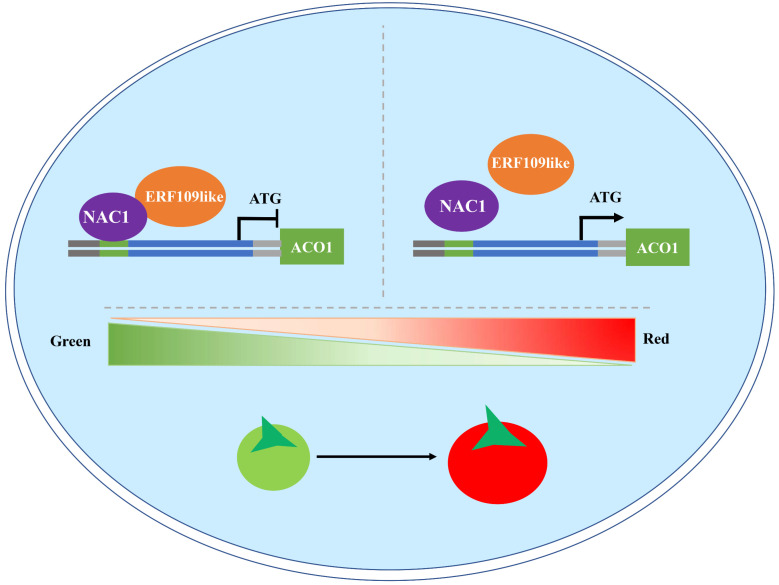
Postulated working model of association of ERF109-like with NAC1 to regulate *ACO1* expression, thereby controlling tomato fruit ripening. At the early stage of tomato fruit ripening, ERF109-like could interact with NAC1 to inhibit *ACO1* transcription, thereby negatively regulating the tomato fruit ripening process. At the late stage of tomato fruit ripening, the interaction between ERF109-like and NAC1 reduced, and the inhibition of *ACO1* was removed, thereby positively regulating the ripening process of tomato fruit. Arrow, positive effect; bar, negative regulation.

The NAC transcription factor, *SlNOR-like1*, could both directly bind to *SlACS2*, *SlACS4*, *SlACS8*, and *SlACO6* ethylene biosynthesis and regulate ABA signal transduction, thereby affecting other ripening-related genes expression to mediate the tomato ripening process [30]. In this study, *SlNAC1*, *SlERF109-like*, and ETH-related genes were highly positively correlated with chlorophyll degradation-related genes, carotenoids accumulation-related genes, and cell wall metabolism-related genes (Figure 10), indicating that SlNAC1-SlERF109-like might indirectly regulate tomato ripening by binding to *ACO1*, and also might directly regulate tomato ripening by affecting ripening-related genes. The tomato fruit ripening process is a complex regulatory network involving multiple activators and repressors, such as ripening inhibitor (*RIN*), non-ripening (*NOR*), colorless non-ripening (*Cnr*) etc.; whether *SlNAC1*-*SlERF109-like* can regulate these marker genes to mediate the ripening process in fruit needs further exploration. In addition, ERFs could also be regulated by upstream *EIN/EILs*, etc. [31,32]. The specific regulatory mechanism of SlNAC1-SlERF109-like remains to be investigated.

## 4. Materials and Methods

### 4.1. Plant Materials Growth 

The ‘*Micro Tom*’ tomato fruits (*Solanum lycopersicum*) were grown in the greenhouse at the School of Food and Biological Engineering (Hefei University of Technology, Hefei, China). The tomato plant growth conditions were 16 h light/8 h dark, and a constant temperature of 25 °C. Generally, the ‘*Micro Tom*’ tomato plant height is 40 cm, and the tomato fruit diameter is 1.5 cm. We planted 50 tomato plants, recorded the time of flowering, and eventually selected 70 tomato fruits which had the same weight, diameter, and growth time for the experiments. The samples of tomato fruit were frozen and grinded in liquid nitrogen quickly, and were stored at −80 °C for subsequent experiments. 

### 4.2. Screening, Phylogenetic Analysis and Data Exploration of Genes in Plants

The ripening-related genes used in this study were screened from the transcriptome data of tomato fruit ripening. The amino acid and coding sequences of ripening-related genes were obtained from the Phytozome public database (https://phytozome-next.jgi.doe.gov/ (accessed on 5 April 2023)). The phylogenetic relationship was analyzed by phylogenetic tree, which was constructed by MEGA 7.0 [33] with the Neighbor-Joining method and 1000 bootstrap replicates. Multiple sequence alignment was performed on multalin (http://multalin.toulouse.inra.fr/multalin/ (accessed on 6 April 2023)). The original expression data of the transcription of *SlERF109-like* in different tomato tissues were obtained from the online tool tomexpress (http://tomexpress.toulouse.inra.fr/ (accessed on 10 May 2023)) query and the tomato cultivar was “*Micro Tom*”.

### 4.3. RT-qPCR

The extraction method used for the total RNA has been previously reported [1]. Total RNA was extracted from 0.1 g after grinding and freezing the tomato fruit samples using the RNA Extraction Kit (Tiangen, Beijing, China), and the reagent for extraction was Trizol. Then, the cDNA was synthesized using a reverse transcription kit (PrimeScript RT Master Mix; Takara, Kyoto, Japan). The cDNA produced by the reverse transcription kit was used for the analysis of gene expression levels using quantitative polymerase chain reaction (qPCR). *Tubulin* and *Actin* tomatoes were used as housekeeping genes for the normalization of data. Primers for the qPCR analysis are listed in Table S1. 

### 4.4. Dual-Luciferase Reporter System Assay

For the dual-luciferase reporter assay, the upstream promoter sequence of SlACO1 (2 kb) was cloned and inserted into pGreen II 0800-LUC, and the coding sequences of transcription factors were cloned and inserted into a pSAK277 vector. The primers are listed in Table S1. Then, pGreen II 0800-LUC-SlACO1 and pSAK277-transcription factors were transformed into *Agrobacterium* strain GV3101 (PM90) with the pSoup helper plasmid. Agrobacterium containing pGreen II 0800-LUC-SlACO1 and pSAK277-transcription factors was expanded to OD_600_ = 0.8 in liquid LB medium, respectively. The method for the dual-luciferase reporter system assay was carried out as described by Yoo et al. (2007) [34]. The ratio of transactivation activities of firefly luciferase and renilla luciferase was determined using the Dual-Luciferase^®^ Reporter Assay System according to the manufacturer’s instructions, (E1910; Promega, Madison, WI, USA).

### 4.5. Luciferase Complementation Assay

The coding sequences of *SlNAC1* and *SlERF109-like* were cloned and inserted into pCAMBIA1300-nLuc (no stop codon) and pCAMBIA1300-cLuc vectors. The primer sequences are listed in Table S1. Agrobacterium containing recombinant plasmids was transformed into *Agrobacterium* strain GV3101 (PM90) with the pSoup helper plasmid and then expanded to OD_600_ = 0.8 and mixed at a ratio of 1:1. The method for the transformation was carried out as described by Chen et al. (2008) [35] and the determination of firefly luciferase activity was performed using a Steady-Glo^®^ Luciferase Assay System (E2510, Promega, Madison, WI, USA).

### 4.6. Yeast One-Hybrid (Y1H) Assay

For the Y1H assay, the promoter of *SlACO1* was analyzed by PlantCare (http://bioinformatics.psb.ugent.be/webtools/plantcare/html/ (accessed on 25 April 2023)), and the 200 to 300 bp fragments containing an NAC binding site or an ERF binding site were cloned and inserted into the pAbAi vector. The coding sequences of *SlNAC1* and *SlERF109-like* were cloned and inserted into the pGADT7 vector. The methods for the self-activation of bait vectors and yeast one-hybrid were carried out as described by Li et al. (2020) [36]. The primers used are listed in Table S1. 

### 4.7. Yeast Two-Hybrid (Y2H) Assay 

The yeast two-hybrid (Y2H) assay was performed according to the method of Li et al. (2020) [36]. The coding sequence of *SlERF109-like* was divided into three segments (S1:1 to 282 bp; S2: 283 to 475 bp; S3: 476 to 669 bp) and inserted S1, S2, and S3, and the full-length of the coding sequence was inserted into the pGBKT7 vector separately to examine the *SlERF109-like* self-activation. The primers are listed in Table S1. Then, pGADT7-*SlNAC1* was co-transformed with pGBKT7-*SlERF109-like*-S1, pGBKT7-*SlERF109-like*-S2, pGBKT7-*SlERF109-like*-S3, and pGBKT7-*SlERF109-like* separately into yeast strain AH109 cells and the interaction between *SlNAC1* and *SlERF109-like* was observed. 

### 4.8. VIGS of SlNAC1 and SlERF109-like in Tomato Fruit

The *SlCAS1* and *SlWRKY71* fragments used for VIGS were analyzed by https://vigs.solgenomics.net/ (accessed on 8 May 2023), before being cloned from the cDNA of ‘*Micro Tom*’ tomato fruits and amplified by TransStart Fast Pfu DNA polymerase (AP221-01, Transgen, Beijing, China) in order to insert them into the pTRV2 vector to generate recombinant pTRV2-*SlNAC1* and pTRV2-*SlERF109-like*. The primers for VIGS are listed in Table S1. Methods for the cultivation of the *A. tumefaciens* strain GV3101 containing the appropriate vectors and transfection of tomato fruits were carried out according to Fantini et al. (2016) [37]. 

### 4.9. Transient Overexpression of SlNAC1 and SlERF109-like in Tomato Fruit

The coding sequences of *SlNAC1* and *SlERF109-like* were cloned and inserted into pSAK277 vectors between the *EcoR*I and *Xho*I restriction sites. The primers are listed in Table S1. The methods for the *A. tumefaciens* strain GV3101 containing the appropriate vectors cultivation and transfection of tomato fruits were performed as described by Yao et al. (2017) [38]. 

### 4.10. Determination of Chlorophyll and Carotenoids Contents in Tomato Fruit

One gram of tomato fruit powder was extracted with acetone and hexane (2:3 volume); afterwards, the quantitative determination of chlorophyll and carotenoids was carried out. The chlorophyll and carotenoids levels were measured and calculated based on the equations described by Nagata and Yamashita (1992) [39].

### 4.11. Statistical Analysis

Data were based on three replicates in each experiment, and the experiments were repeated independently three times. Statistical significance was assayed using a one-way analysis of variance with IBM SPSS Statistics (SPSS version 20.0; Armonk, NY, USA), and the results are expressed as the means ± SDs. Significant differences were calculated using a *t*-test (*p* < 0.01 or *p* < 0.05). 

## 5. Conclusions

Overall, *SlNAC1* and *SlERF109-like* were identified as playing a negative role in the tomato ripening process by way of the transient silencing and over-expression of genes. And SlERF109-like could form with SlNAC1 to be a regulatory complex that inhibits *SlACO1* transcription. Therefore, we provide a new model showing that a regulatory complex—SlNAC1-SlERF109-like—could bind to promoter *ACO1* and down-regulate the key ETH biosynthesis gene, *ACO1*, delaying the tomato ripening process at multiple levels, and offer a theoretical basis for the precise regulation of ripening and senescence in tomato fruit.

## Data Availability

Data are contained within the article and Supplementary Materials.

## Supplementary Materials

The supporting information can be downloaded at: https://www.mdpi.com/article/10.3390/ijms25031873/s1.

## Abbreviations

ETH, ethylene; TFs, transcription factors; VIGS, Virus induced gene silencing; RT-qPCR, quantitative reverse transcription polymerase chain reaction; Y1H, Yeast one-hybrid; Y2H, Yeast two-hybrid.
