# Supplementary material for: SlERF109-like and SlNAC1 Coordinately Regulated Tomato Ripening by Inhibiting ACO1 Transcription
Source: Int J Mol Sci. 2024 Feb 3;25(3):1873. doi: 10.3390/ijms25031873 (PMC10855853; doi:10.3390/ijms25031873)
Supplement: Supplementary file 1 [file ijms-25-01873-s001.zip › ijms-2807061-supplementary.pdf]

Table S1. Primers used in the present study.

**Primers**	Sequences	ID
SlERFTINY-pSAK277-F	ACTAGTGGATCCAAAGAATTCATGTCTACTACACATCGCTATAAAAACG	Solyc03g120840
SlERFTINY-pSAK277-R	TCATTAAAGCAGGACTCTAGATAATATTAAACATATTTCTTCTATAATCAAATGTATAA	
SlERF109-like-pSAK277-F	ACTAGTGGATCCAAAGAATTCATGCAGAGGTCAACAAAACTTAGTG	Solyc10g050970
SlERF109-like-pSAK277-R	TCATTAAAGCAGGACTCTAGACTAGAAGCTATGAGCAATATAGCCATT	
SlNAC73-pSAK277-F	ACTAGTGGATCCAAAGAATTCATGGCATGGTGCAGTCAATCA	Solyc02g077610
SlNAC73-pSAK277-R	TCATTAAAGCAGGACTCTAGATCACTTCTCTTTGCTTGCTTCTGA	
SlNAC35-pSAK277-F	ACTAGTGGATCCAAAGAATTCATGGAAGATCTACCAGGGTTCCG	Solyc10g055760
SlNAC35-pSAK277-R	TCATTAAAGCAGGACTCTAGACTAAAAGTTATGCACAAACGCGG	
SlNAC1-pSAK277-F	ACTAGTGGATCCAAAGAATTCATGAACAAAGGAGCAAACGGAA	Solyc04g009440
SlNAC1-pSAK277-R	TCATTAAAGCAGGACTCTAGATTAGTAAGGTTTTTGCATGTATAGGAAC	
SlWRKY-IID-pSAK277-F	ACTAGTGGATCCAAAGAATTCATGGCTGTGGAGCTAATGATGG	Solyc02g093050
SlWRKY-IID-pSAK277-R	TCATTAAAGCAGGACTCTAGATTAAGAAGACTCTAAAATGAGACTACTTGTTT	
SlWRKY51-pSAK277-F	ACTAGTGGATCCAAAGAATTCATGGAAAATTTTCCCTATAGCTCATC	Solyc04g051690
SlWRKY51-pSAK277-R	TCATTAAAGCAGGACTCTAGACTAAAGGTGAAGATTGTGAAGGGC	
SlWRKY65-pSAK277-F	ACTAGTGGATCCAAAGAATTCATGGAAGATAGTTCATACAAAAATCTATTTT	Solyc07g055280
SlWRKY65-pSAK277-R	TCATTAAAGCAGGACTCTAGATTATCCTGTACCTCCGCAGCA	
SlWRKY71-pSAK277-F	ACTAGTGGATCCAAAGAATTCATGGGTGATGAACTAAGAGATTTGTACT	Solyc02g071130
SlWRKY71-pSAK277-R	TCATTAAAGCAGGACTCTAGATCATGGTTCTTGTTTAAGATTAAACATAG	
SlACO1- pGreen II 0800-LUC-F	GTCGACGGTATCGATAAGCTTCAATTAAATCTCATCACCCCAACC	Solyc07g049530
SlACO1- pGreen II 0800-LUC-R	CGCTCTAGAACTAGTGGATCCAGACACATGTAGGATATTTTTAAATTATTTG	
SlERF109-like-Nluc-F	CGAGCTCGGTACCCGGGATCCATGCAGAGGTCAACAAAACT	

SlERF109-like-Nluc-R	CGCGTACGAGATCTGGTCGACGCTATGAGCAATATAGCCATTAGC
SlERF109-like-Cluc-F	CCGGGGCGGTACCCGGGATCCATGCAGAGGTCAACAAAACT
SlERF109-like-Cluc-R	ACGAAAGCTCTGCAGGTCGACCTAGAAGCTATGAGCAATATAGCC
SlNAC1-Nluc-F	CGAGCTCGGTACCCGGGATCCATGAACAAAGGAGCAAACGG
SlNAC1-Nluc-R	CGCGTACGAGATCTGGTCGACGTAAGGTTTTTGCATGTATAGG
SlNAC1-Cluc-F	CCGGGGCGGTACCCGGGATCCATGAACAAAGGAGCAAACGG
SlNAC1-Cluc-R	ACGAAAGCTCTGCAGGTCGACTTAGTAAGGTTTTTGCATGTATAG
ACO1-F1-pAbAi-F	AAATGATGAATTGAAAAGCTTAAGAGAAAAGTTGAAAACACCCATAA
ACO1-F1-pAbAi-R	ATACAGAGCACATGCCTCGAGGACTTATTAGTTTATTTTTCGAACTATTGATAG
ACO1-F2-pAbAi-F	AAATGATGAATTGAAAAGCTTGTTAATTGGGCTACGAAAGATTCG
ACO1-F2-pAbAi-R-	ATACAGAGCACATGCCTCGAGTCACATACCATAGTATTCATAGTTGTGGG
SlNAC1-ADT7-F	GCCATGGAGGCCAGTGAATTCATGAACAAAGGAGCAAACGGAA
SlNAC1-ADT7-R	ACGATTCATCTGCAGCTCGAGTTAGTAAGGTTTTTGCATGTATAGGAAC
SlERF109-like-ADT7-F	GCCATGGAGGCCAGTGAATTCATGCAGAGGTCAACAAAACTTAGTG
SlERF109-like-ADT7-R	ACGATTCATCTGCAGCTCGAGGAAGCTATGAGCAATATAGCCATTAGC
SlERF109-like-S1-BKT7-F	TCAGAGGAGGACCTGCATATGATGCAGAGGTCAACAAAACTTAGTG
SlERF109-like-S1-BKT7-F	CTAGTTATGCGGCCGCTGCAGCTTCTTCTTCTTTTTCGTCTTCCC
SlERF109-like-S2-BKT7-F	TCAGAGGAGGACCTGCATATGAATTACAGGGGAGTGAGACAGAGAC
SlERF109-like-S2-BKT7-F	CTAGTTATGCGGCCGCTGCAGTGAAGTGTAATCTGTAAATGAGAAATTAAGC
SlERF109-like-S3-BKT7-F	TCAGAGGAGGACCTGCATATGATTCAACAACACAATACTACTACACCCA
SlERF109-like-S3-BKT7-F	CTAGTTATGCGGCCGCTGCAGGAAGCTATGAGCAATATAGCCATTAGC
SlERF109-like-BKT7-F	GCCATGGAGGCCAGTGAATTCATGCAGAGGTCAACAAAACTTAGTG

SlERF109-like-BKT7-F	CTAGTTATGCGGCCGCTGCAGGAAGCTATGAGCAATATAGC CATTAGC	
SlNAC1-VIGS-F	CGTGAGCTCGGTACCGGATCCGTACCGCCTCGCCAACGT	
SlNAC1-VIGS-R	GTGAGTAAGGTTACCGAATTCACCATCCAAATAGTTTAGCT GAAAATC	
SlERF109-like-VIGS-F	CGTGAGCTCGGTACCGGATCCGAAGAAGAAGAATTACAG GGGAGTG	
SlERF109-like-VIGS-R	GTGAGTAAGGTTACCGAATTCCTAGAAGCTATGAGCAATAT AGCCATT	
SlERF109-like (forward)	GGAGTGAGACAGAGACCATG	
SlERF109-like (reverse)	ATGTTCCAAGCCATACTCTAGC	
SlTubulin (forward)	TAGAGCCTGGTACGATGGATAG	Solyc08g006890
SlTubulin (reverse)	CAACTCAGCGCCTTCAGTATAA	
SlActin (forward)	GAGAAGCACATTCCCTGAAAG	Solyc04g011500
SlActin (forward)	AGAACTCCACCATCACCACC	
SlNAC1 (forward)	TCGATGACTAGAATGCACACG	
SlNAC1 (reverse)	TCAAGGGTGTTTCTGTGGTC	
SlNAC73 (forward)	AAGGATGGTCAAGTGCGTC	
SlNAC73 (reverse)	ACCTGTCTTGTGCCATCTTG	
SlNAC35 (forward)	GAAATGAAGCACGTACACAGC	
SlNAC35 (reverse)	CTTCATCCTTGCTGATTGACG	
SlWRKY-IID (forward)	GTGTATTCTCCAACTCCGATCC	
SlWRKY-IID (reverse)	TCTGTTTCCCCTGTTAACGAC	
SlWRKY51 (forward)	AGAGTTGGAGGTGTTGGATG	
SlWRKY51 (reverse)	ATCTTTATTGTCCCTTTCTACTCTTTTC	
SlWRKY65 (forward)	GAACACAATCACCAAATCCCG	
SlWRKY65 (reverse)	GTAGAGGTAGTGGGTGAACTG	
SlWRKY71 (forward)	TCATCTACCAGCTACTCTCCG	
SlWRKY71 (reverse)	GGCATGTCCATAAAATTGAGGC	
SlERFTINY (forward)	GGAGTTAGGATGAGAAGTTGGG	
SlERFTINY (reverse)	TCCTTTTATACTCACCGCTGC	
SlACO1(forward)	CCATGTCCTAAGCCCGATTT	Solyc07g049530
SlACO1 (reverse)	GGCCACTCACTTTGTCATCT	
SlSGR1 (forward)	GGCTATCTCCCAAACCATCAA	Solyc08g080090
SlSGR1 (reverse)	ACTCTGCAACAACTTCATCTCT	
SlPPH (forward)	CAGATGTCGTGGATGGGAAA	Solyc01g088090
SlPPH (reverse)	GCCTTTAGCTTCACTTGGTAAAC	
SlPAO (forward)	CCTCATCGTCTTGCTCCTTTAT	Solyc11g066440
SlPAO (reverse)	GCAGCTTGAGGTATCCTTGT	
SlNYC1 (forward)	GATCAGGGAAGAGCACTGTATG	Solyc07g024000
SlNYC1 (reverse)	GAGAACACAGACACCCAAGTAT	
SlPSY1 (forward)	GAAGATGCCAGAAGAGGAAGAG	Solyc03g031860
SlPSY1 (reverse)	GGTCACCCTTCCAGCAAATA	
SlPDS (forward)	CAAGACCAGAGCTGGACAATAC	Solyc03g123760

SlPDS(reverse)	CAAACCTGCACCAGCAATAAC	
SlZDS (forward)	GATTGGTTCCTCAGAAGTGGAG	Solyc01g097810
SlZDS (reverse)	CCAGCCATTGTAGCGTAGTT	
SlE4 (forward)	CATGACCCGACCACTCTAAATC	Solyc03g111720
SlE4 (reverse)	CAGTTGAGCCTGAGCATCAT	
SlE8 (forward)	GTCTCGGTCTCGATCGTAGTTA	Solyc09g089580
SlE8 (reverse)	AGGACATGGTGGGTAGTAGTT	
SlPG (forward)	TTGGAGGAGGAGGAACTATCA	Solyc10g080210
SlPG (reverse)	CCTGCATGGCAGTGATTTATTT	
SlCEL2 (forward)	CTGCTGTTTGCCCTTTCTATTG	Solyc09g010210
SlCEL2 (reverse)	TGCCTTCTTCTTGTTGCTTTATG	
SlEXP (forward)	CCGACGATTGGACACCTAAA	Solyc06g051800
SlEXP (reverse)	GGTCCTCCTTAATCAAAGGACATA	
SlTBG4 (forward)	GCCATTGGCCTGGATACATA	Solyc12g008840
SlTBG4 (reverse)	AGAAGGTTGTCCGCAGTTAG	
SlXTH5 (forward)	AGGATTCAGCCATCTCTTTGG	Solyc01g081060
SlXTH5 (reverse)	ACTTGAACCCTGAACCTGTG	
